# Reply to Shaikh et al. Comment on “Rochmah et al. Economic Burden of Stroke Disease: A Systematic Review. *Int. J. Environ. Res. Public Health* 2021, *18*, 7552”

**DOI:** 10.3390/ijerph19074261

**Published:** 2022-04-02

**Authors:** Thinni Nurul Rochmah, Indana Tri Rahmawati, Maznah Dahlui, Wasis Budiarto, Nabilah Bilqis

**Affiliations:** 1 Department of Health Administration and Policy, Faculty of Public Health, Universitas Airlangga, Surabaya 60115, Indonesia; indana.tri.rahmawati-2018@fkm.unair.ac.id (I.T.R.); maznahd@ummc.edu.my (M.D.); wasisbudiarto@fkm.unair.ac.id (W.B.); nabilahbilqis@gmail.com (N.B.); 2 The Airlangga Centre for Health Policy Research Group, Surabaya 60115, Indonesia; 3 Centre of Population Health, Department of Social and Preventive Medicine, Faculty of Medicine, University of Malaya, Kuala Lumpur 50603, Malaysia

Thank you for being interested in making a review [1] of our article [2]. We really appreciate and appreciate the experts who have provided input to the studies that we have done. We will provide some responses (in italics) to the reviews that have been given as follows:

The authors followed the PRISMA methodology for systematic review but have not excluded any type of study designs as stated. Surprisingly, there are no exclusion criteria, and sufficient reasons were not provided in this regard.

In this systematic review, we followed the PECOS framework (participant or population, exposure, context, and outcome measures) and had listed the inclusion criteria with enough keywords so that we would not miss articles that are related to economic evaluation of stroke management. We observed that articles on economic evaluation of disease management use various methods and present the economic evaluation findings in various forms, such as different cost perspectives, different types of cost—direct or indirect, and variation in the inclusiveness of cost (procedure, activity or total management cost). We wanted to capture any cost in relation to stroke management and thus purposely did not list exclusion criteria. We believed that without including exclusion criteria, we could get a more extensive and complete (comprehensive) search of relevant articles. The comprehensive results enabled us to provide an overview for the mapping of the various study designs, costing methods, cost inclusiveness, cost perspectives and other economic outcome of stroke management. We realize that our explanation in the exclusion criteria section is lacking which may have led to this misunderstanding.

Authors included all types of publications, study designs including prospective, retrospective, database analysis, mathematical models, surveys, and COI studies published between the year 2015–2020 in the English language examining the economic burden (direct and indirect costs) in the stroke population. The admixture of all types of study designs might have enabled the authors to extract similar characteristics but narrowing it down to a distinct inference was obscure.

Our article aimed to conduct a systematic review of the economic burden of stroke. In the field of economic burden, there are several study designs used, including prospective cost studies, retrospective cost studies, database analysis, mathematical models, surveys, and COI studies. We included all these methods in the inclusion criteria in the hope that we could obtain a more complete article, and to provide material for analysis related to the weaknesses and strengths of each study design. However, in the end, we did not find many study designs (only prospective cost studies, retrospective cost studies, and cross-sectional/surveys).

In our study, we attempted to describe the findings based on methodological characteristics and estimates of the economic burden of stroke. With the findings presented descriptively, we aimed to perform an overview/mapping of the economic burden due to stroke based on the results of each of the included studies.

We feel the exclusion could have been based on specific economic evaluation indicators (especially the QALY and DALY) as mentioned by the authors in the introduction.

QALYs and DALYs on their own are only measuring the disease burden whereby they combine the quality of life or disability with duration of illness. DALY is an important attribute in the study of disease burden in the context of the Global Burden of Disease. QALYs and DALYs applied in economic evaluation have been commonly used in justifying the adoption of health interventions. The ratios of Cost per QALY gained or Cost per DALY avoided from a cost-effectiveness analysis (CEA) are commonly used to identify which health intervention for the same objective would be more cost-effective. This systematic review aimed to determine the cost of disease (stroke) management. Including QALYs and DALYs would not meet our paper’s objective and similarly, including the cost per QALY gained or cost per DALY avoided would not necessarily provide the details of the cost incurred for stroke management. We also did not listed QALYs and DALYs in the exclusion criteria because some CEA studies may present the various costs incurred in managing stroke as input variables in their CEA model.

A well-defined exclusion criterion enables the researcher to eliminate biases arising due to the missing outcome data

We greatly appreciate the input on the importance of exclusion criteria to minimize bias in the results of a systematic review. In our systematic review, we extracted descriptive data based on methodological characteristics and estimates of the economic burden due to stroke from each included article. It is hoped that a more comprehensive picture/mapping of the economic burden due to stroke can be obtained based on the various characteristics of the existing studies. The authors’ input can be an important note for future research

Concerning the methodology, the assessment of 2814 articles for full-text screening can be an exhaustive task. The use of suitable software for screening abstracts and removing duplicates instead of Microsoft Excel thereby reducing the numbers for scrutiny with a low risk of manual error could have helped in the process.

We greatly appreciate the authors’ input on using suitable software for screening abstracts and removing duplicates. Indeed, in this systematic review, we have not used the software. But of-course it would be very beneficial for future research.

Also, justification for excluding titles and abstracts was lacking, most likely due to the absence of stringent eligibility criteria.

A total of 2720 titles and abstracts were excluded from the search for articles because they were not related to the research theme, namely the economic burden of disease due to stroke. The majority of articles were not included because:

a. The study was on economic burdens other than stroke (e.g., cardiovascular in general, not specifically on stroke, diseases unrelated to the cardiovascular group)
b. The study was on other aspects of stroke and did not examine the cost related to stroke management.

We reviewed George’s study, thereby noting that the above method definitely aided in extracting the economic and epidemiological variables for systematic evaluation but failed to evaluate the overall quality of the included study. We suggest the inculcation of a valid and robust qualitative assessment tool according to the types of study designs. To enhance the quality, the risk of bias assessment tool helps in identifying the critical domains and sources of bias such as selection bias, attrition bias, reporting bias amongst others.

In the research by George it is stated that “We appraised the quality of included studies using a seven-question quality assessment tool developed for the purpose of this study. The tool focused on two aspects: the design of the economic study, inspired by the CHEERS (Consolidated Health Economic Evaluation Reporting Standards) Statement; and, if applicable, the design of the epidemiological study alongside which the economic study was conducted.” Based on this, we decided to adopt the ‘7 question quality assessment tool’ because we think that the objectives of our research are similar to those of George’s research [3].

We can use the various quality assessment appraisal tools available that are specifically made for one design study, but we did not use them considering the similarity of the study we carried out with George’s. We are grateful for the advice of the reviewers as this is a limitation By neglecting any one sector of the society especially the ‘*lower-middle*’ and ‘*low-income*’ regions or countries, policy enactment takes a backseat and happens to be biased. It is interesting to note that economic burden has been studied in ‘*high income*’ and ‘*upper-middle income*’ countries. The major pitfall of the review is that not a single study got extracted in the included articles on ‘*lower-middle-income countries*’ or ‘*low-income*’ countries. This probably could have been due to the absence of stringent eligibility criteria and a possibility of a meta-analysis could have arisen.

We are aware that the articles that we found did not cover all categories of country economic status based on the classification of country income by the World Bank (Lower-Middle Income Economies, Low-Income Economies, Upper-Middle Income Economies, High-Income Economies). In the included articles, only research results were obtained in countries that are included in upper-middle income economies and high-income e. We did not state explicitly in the inclusion criteria any specifics about the country’s income classification. We decided to classify the research setting based on the classification of country income by the World Bank to minimize bias and gaps in research results in each of the articles included.

In addition, the absence of articles from countries categorized as lower-middle-income countries and low-income countries may also indicate there is still only limited research on the economic burden of stroke in these countries.

## Data Availability

This study didn’t report any data.
